# Rib fracture secondary to cough‐induced trauma

**DOI:** 10.1002/ccr3.8823

**Published:** 2024-04-26

**Authors:** Ahmed Qasim Mohammed Alhatemi, Hashim Talib Hashim, Ali Talib Hashim

**Affiliations:** ^1^ Department of Internal Medicine Al Nasiriyah Teaching Hospital Thi Qar Iraq; ^2^ Research Department Warith Al Anbiyaa University Karbala Iraq; ^3^ Golestan University of Medical Sciences Gorgan Iran

**Keywords:** imaging and radiology, orthopaedics, respiratory medicine

## Abstract

Early identification of rib fractures, even in young patients without chronic diseases, is essential. Prompt diagnosis facilitates appropriate management, aiding in pain control and addressing underlying causes such as persistent coughing. Additionally, vigilance for complications such as pneumothorax and rib displacement is crucial for optimizing patient care.

## Graphical abstract

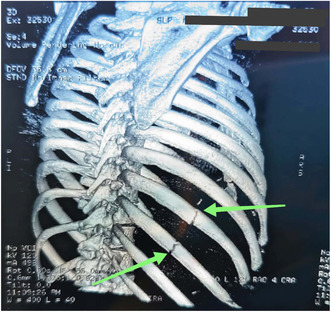

## 1 CASE PRESENTATION

**FIGURE 1:**
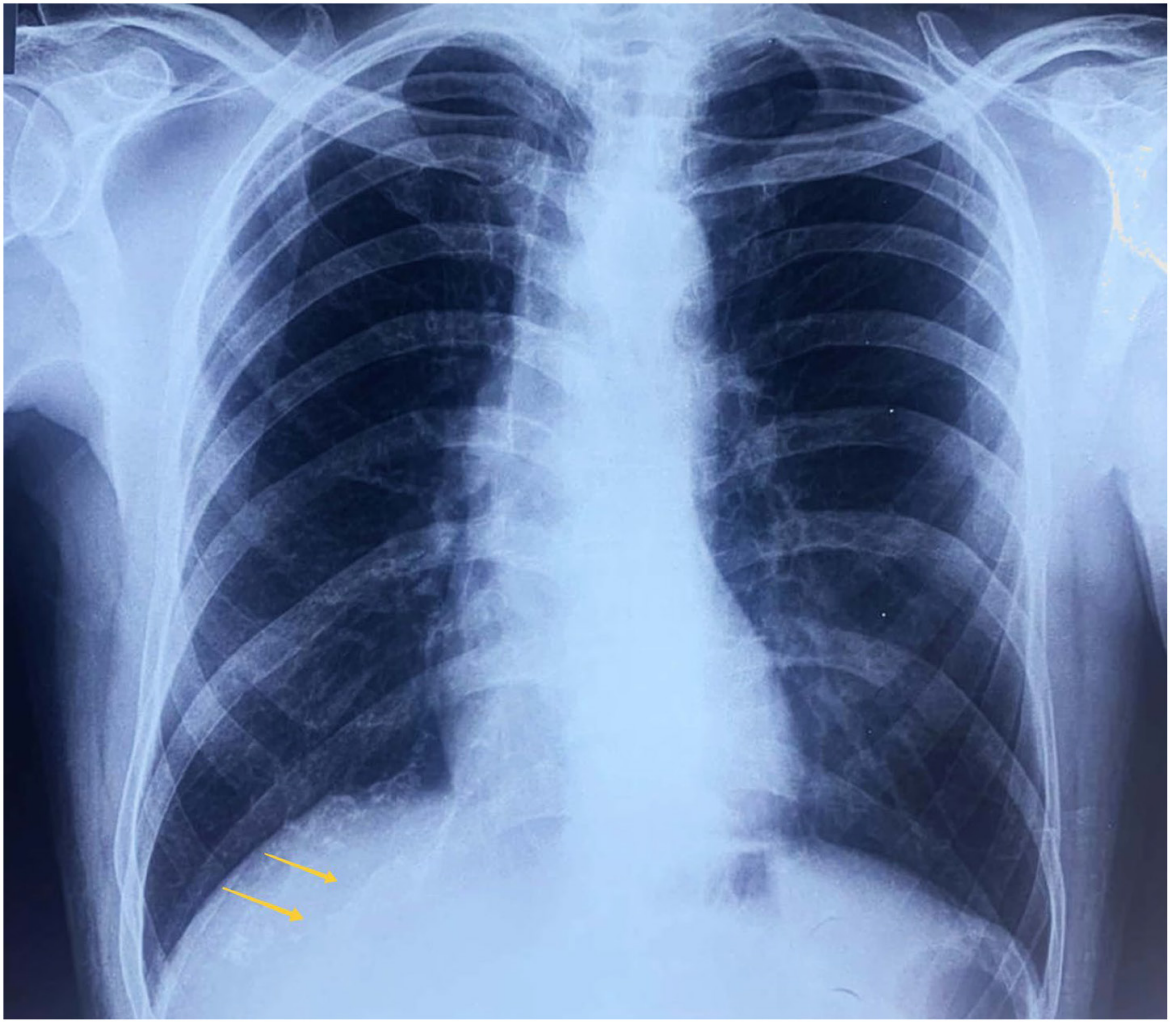
The chest X‐ray PA view is labeled as normal, with yellow arrows indicating the suspected fracture site.

A 26‐year‐old male presented to our hospital with a 2‐week history of ongoing chest pain exacerbated by coughing, sneezing, or laughing. He reported a mild episode of bronchitis 1 month ago, lasting 3 weeks, followed by the development of constant, dry, non‐productive cough and chest pain. Past medical history revealed no chest trauma, chronic diseases, or medication allergies. The patient, a builder, and a smoker of 20 packs per year denied alcohol consumption and did not take any medications such as steroids nor immunosuppressive drugs for chronic or acute conditions. There was no family history of similar conditions. Physical examination showed tenderness upon palpation of the chest, particularly in the right lower posterior chest wall, with no crackles, wheezing, or barrel chest noted. His pulse was slightly elevated at 92 beats/min due to pain, blood pressure was 127/81 mmHg, oxygen saturation at 92% on room air, respiratory rate at 21 cycles/min, and body temperature was normal at 36.7°C. Laboratory investigations, including a complete blood count and metabolic panel, were unremarkable. His chest X‐ray was unremarkable (Figure 1).

## 2 WHAT IS YOUR DIAGNOSIS?

**FIGURE 2:**
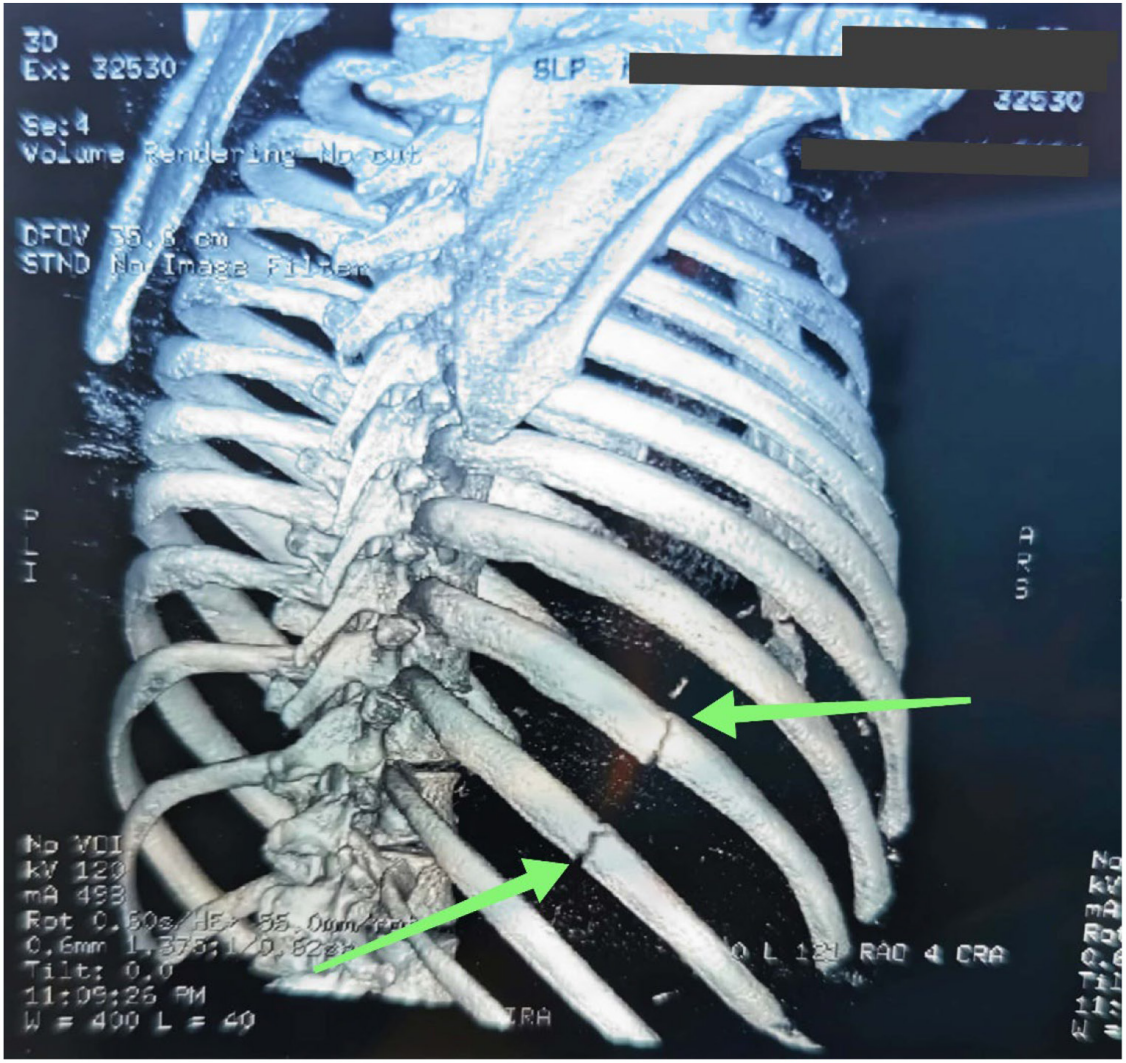
Chest CT 3D reconstruction showing a linear fracture of the right ninth and tenth ribs (green arrows).

Costochondritis and pleurisy were at the top of the deferential diagnosis, alongside rib fracture due to localized chest wall tenderness. We proceeded with a chest CT scan, which revealed linear fractures of the ninth and tenth ribs. The diagnosis in this case is a cough‐induced rib fracture, as shown in Figure 2. Parathyroid hormone, blood calcium, and serum protein electrophoresis were performed to rule out secondary etiologies of pathological rib fractures such as hyperparathyroidism and multiple myeloma, although they are rare under 40, but were unchanged. Given the association between cough‐induced rib fractures and decreased bone density,^1^ a dual‐energy X‐ray absorptiometry (DEXA) scan was scheduled. The patient was managed with antitussives and nonsteroidal anti‐inflammatory drugs. For his chest pain, the patient was administered simple analgesia consisting of codeine phosphate and acetaminophen, with tramadol if needed. Additionally, lifestyle modifications were advised, including smoking cessation.

## 3 DISCUSSION

Non‐traumatic rib fracture typically results from factors like osteoporosis, medication use, and persistent coughing. Osteoporosis weakens bones, medications like corticosteroids can contribute, and persistent coughing places stress on ribs, leading to fractures.^2^

Muscles contributing to rib fractures include the external and internal intercostal muscles, diaphragm, serratus anterior, rectus abdominis, and interchondral muscles.^3^

There is no a clear male or female predominance in rib fractures, though some studies suggest a slightly higher incidence in males due to occupational and recreational activities, while women may experience fractures more frequently due to osteoporosis after menopause.^4^

Ribs most likely to be fractured are the middle ribs (third to ninth ribs) due to their relatively less flexibility compared to upper and lower ribs. Typically, one or two ribs are fractured, but in some cases, multiple ribs may be involved, especially in severe trauma.^5^

The diagnosis should not be limited to the results of a conventional radiograph; instead, the patient's history and symptoms need to be considered, and the patient's presentation needs to be continuously monitored for changes in the clinical presentation for appropriate management.^6^ Clinical examination, including physical examination tests, is crucial for detecting emergency etiologies and narrowing the differential diagnosis.^7^

Rib fractures are often difficult to identify on plain film imaging due to subtle fracture lines, overlying soft tissues obscuring visualization, incomplete views, superimposition of structures, and variations in rib orientation, resulting in variable accuracy. Studies have shown that plain radiographs may miss up to 50% of rib fractures,^5^ necessitating additional imaging for accurate diagnosis, such as a chest CT scan. CT scans are more sensitive than plain radiographs for detecting rib fractures and can also provide information regarding the number of ribs involved.^8^

Management focuses on pain control, respiratory support, and addressing the underlying cough. Though considered rare, healthcare professionals must be vigilant, recognizing the potential severity of this complication and tailoring interventions to ensure optimal patient outcomes. Understanding and addressing cough‐induced rib fractures contribute to comprehensive patient care.^9^

The correlation between the number of ribs fractured and mortality, especially in older patients, is well‐established. Studies have shown that an increased number of fractured ribs is associated with higher mortality rates, particularly in older individuals. Older patients often have decreased bone density due to conditions like osteoporosis, making their ribs more susceptible to fractures even with minor trauma.^10^

Complications of rib fractures include pneumothorax, hemothorax, pulmonary contusion, and rib displacement leading to lung injury, managed through pain control, respiratory support if necessary, chest physiotherapy, rib fracture stabilization with splinting or bracing, and monitoring for and treating complications such as pneumothorax or hemothorax.^11^

## AUTHOR CONTRIBUTIONS

**Ahmed Qasim Mohammed Alhatemi:** Conceptualization; data curation; writing – original draft. **Hashim Talib Hashim:** Investigation; methodology; writing – review and editing. **Ali Talib Hashim:** Project administration; validation; visualization.

## FUNDING INFORMATION

No source of funding was received.

## CONFLICT OF INTEREST STATEMENT

The authors declare that they have no competing interests.

## CONSENT

Written informed consent was obtained from the patient to publish this report in accordance with the journal's patient consent policy.

## Data Availability

The data that support the findings of this study are available from the corresponding author upon reasonable request.
